# Risk assessment of metals measured in regulated Canadian dried cannabis and cannabis vaping products: case study and perspectives

**DOI:** 10.3389/ftox.2025.1755875

**Published:** 2026-01-15

**Authors:** Sathish Achuthan, Andrew Waye, Hanan Abramovici

**Affiliations:** Office of Cannabis Science and Surveillance, Controlled Substances and Cannabis Branch, Health Canada, Ottawa, ON, Canada

**Keywords:** cannabis vaping, dried cannabis, good production practices, heavy metals, permitted daily exposure (PDE), risk assessment

## Abstract

In 2018, the *Cannabis Act* and its regulations established a strict legal framework for controlling production, distribution, sale and possession of cannabis across Canada. At that time, smoking dried cannabis was the most prevalent mode of consumption, and remains so to date, but cannabis vaping products have become increasingly popular since they were made commercially available in late 2019. Heavy metals are a recognized class of impurities in cannabis products that can pose consumer health concerns. The *Cannabis Regulations* ensure a quality-controlled supply of cannabis by requiring good production practices (GPPs) and refer to pharmacopoeias for impurity tolerance limits. For elemental impurities, pharmacopoeias specify tolerance limits based on route of exposure and as a permitted daily exposure (PDE). This paper presents a risk assessment case study based on levels of metals measured in legal products that have been published by Health Canada on dried cannabis and cannabis vaping liquids to illustrate the challenges in assessing risks from a regulatory and quality control perspective, using daily or almost daily typical (50th percentile) and heavy (95th percentile) use as a worst case scenario. Applying PDEs from established pharmaceutical quality control standards for the newly legalized cannabis industry has its own challenges, characterized by existing uncertainties which must be addressed, in particular as they relate to exposure characterization. This risk assessment identifies that there is a low risk to health from heavy metals in Canadian legal and regulated inhaled cannabis products, especially as most cannabis consumers in Canada are not daily or almost daily users. These findings suggest that the goal of the *Cannabis Act* to mitigate risks to health by providing access to a quality-controlled supply of cannabis has been achieved in this regard.

## 1 Introduction

Cannabis was legalized in Canada on October 17, 2018, marking a new era in the Government’s approach to cannabis control. One year later, the sale of cannabis edibles, extracts and topicals was permitted, and regulations were amended to include new controls to address the public health and safety risks associated with these products.

In Canada, while producers must be compliant with outcome-based requirements for their cannabis products, no pre-market review or approval is required prior to sale. As such, human health risk assessments (HRAs) are often warranted based on signals from reported adverse reactions or from post-market product testing and surveillance. These HRAs establish a health risk type (Health Canada, 2019a) and support risk managers in taking appropriate action to ensure identified risks to consumers can be mitigated, when necessary. However, often the information necessary to assess the risk of an identified hazard with a high degree of certainty is limited or unavailable, posing challenges to cannabis risk assessors.

Cannabis may be consumed in a variety of ways. In Canada, the most prevalent modes of consumption are smoking, oral ingestion and vaping. The most recent Canadian Cannabis Survey (CCS) data showed that the products with the highest proportion of daily/almost daily consumers were dried cannabis (28%, which includes pre-rolled joints and loose dried flower), followed by vape pens/cartridges (21%) (Government of Canada, 2024).

Heavy metals are a recognized class of contaminants in inhaled cannabis products that can be of concern to consumers. These elemental impurities include cadmium and arsenic, that volatilize during pyrolysis and can result in increased toxic and carcinogenic effects (Dryburgh et al., 2018; Lorenz et al., 1989). Another concern is chronic inhalation exposure to elevated levels of metals from any source, including from vaping products, which can have various health consequences, including inflammation, sensitization, toxicity and cancer (Pappas, 2011). Potential sources of metal impurities in inhaled cannabis products include: accumulation in the plant during cultivation; contamination during extraction and processing (Thomas and Destefano, 2024); consumption of product accessories such as rolling papers (Wright et al., 2024); and leaching from vaping device components (Gajdosechova et al., 2022; Gajdosechova et al., 2025; McDaniel et al., 2021).

The *Cannabis Act* aims to protect public health and safety, including by providing access to a quality-controlled supply of cannabis. The *Cannabis Regulations* establish good production practices (GPPs), which require testing for chemical contaminants, including elemental impurities. Contaminant levels must meet generally accepted tolerance limits, appropriate for the intended use of the cannabis product, found in standards such as U.S. Pharmacopeia (USP) and European Pharmacopoeia (Ph Eur) (Health Canada, 2019b). GPPs also require that contaminant testing occurs after the final step in production where contaminants could be introduced or concentrated.

The limits in the USP and Ph Eur standards for elemental impurities are presented as permitted daily exposures (PDEs). PDEs establish absolute levels of the metal (micrograms per day) that should not be exceeded from product use. From the PDEs, as well as from the levels that GPPs can reasonably achieve, concentration limits can be determined and applied as testing specifications. At this time, to the best of our knowledge, the cannabis industry commonly tests for the four toxic metals found in Class 1 of the USP and Ph Eur elemental impurities standards: arsenic (As), cadmium (Cd), mercury (Hg) and lead (Pb). The industry often applies specifications corresponding to concentration limits listed in the pharmacopoeias where no more than 10 g of the product would be consumed per day. These concentration limits (As – 0.2 μg/g; Cd – 0.3 μg/g; Hg – 0.1 μg/g; Pb – 0.5 μg/g) are understood as being readily achievable by GPPs in dried cannabis and cannabis vaping products and are adopted in many US state regulations.

Since legalization, inhaled cannabis products have been identified in the Canadian market that contain other metals at levels above the concentration limits listed in the pharmacopoeias. In such cases, risk analysis helps determine whether a PDE could be exceeded and if action is required to mitigate health risk from reasonably foreseeable use of the product.

There are innumerable challenges in science-based risk decision-making, and this perspective aims to reflect those regarding heavy metals in inhaled cannabis products. For illustrative purposes, we present a case study of a risk analysis conducted on metal impurities identified in dried cannabis and cannabis vaping products.

## 2 Case study

This risk assessment case study was based on data on legal cannabis products from two Health Canada studies. The first was Gajdosechova et al. (2022) where metal particles were reported in unused cannabis vape cartridges (20 products). The second was Health Canada’s Cannabis Data Gathering Program study (Health Canada, 2025), which generated baseline data on heavy metals in dried cannabis (50 products).

The 2023 and 2024 CCS results (Government of Canada, 2023; Government of Canada, 2024) were used to identify appropriate, reasonably foreseeable, consumption amounts to use in the risk assessment (Supplementary Materials S1). Daily or almost daily consumers of dried cannabis who used for medical purposes reported consuming the highest amounts, at 1.5 g/day for typical (median), and 7 g/day for heavy (95th percentile) consumption, so these values were used for the risk assessment for dried cannabis (Table 1). Those who reported using dried cannabis daily/almost daily for non-medical purposes reported using slightly lower amounts, at a 1 g/day median, and 5/g day 95th percentile. Our analysis for dried cannabis was limited to smoking. Dried cannabis is also used in dry herb vapourizers, which is not assessed here, as this use-scenario is more poorly characterized and smoking is the more common mode of consumption. Limited evidence suggests metal transfer from dried cannabis when vapourized is very low (Wang et al., 2024; Cabecinha et al., 2025).

**TABLE 1:** Estimated daily exposure to metals from typical or heavy daily/almost daily dried cannabis use. Shaded cells indicate where a European Pharmacopoeia permitted daily exposure would be exceeded.

Metals	Reported metal concentrations^a^ (µg/g)		Estimated daily exposure (µg/d)				Daily exposure limits^d^ (µg/d)
​	Median	Maximum	Typical^b^ daily use (1.5 g) with median metal concentration	Typical^b^ daily use (1.5 g) with maximum metal concentration	Heavy^c^ use (7.0 g) with median metal concentration	Heavy^c^ use (7.0 g) with maximum metal concentration	
Ag	<0.07	<0.07	<0.105	<0.105	<0.49	<0.49	7
As	<0.02	0.16	<0.03	0.24	<0.14	1.12	2
Au	<0.01	<0.01	<0.015	<0.015	<0.07	<0.07	1
Ba	<3.00	40.68	<4.5	61.02	<21	284.76	300
Cd	<0.03	0.16	<0.045	0.24	<0.21	1.12	3
Co	<0.03	0.12	<0.045	0.18	<0.21	0.84	3
Cr	0.05	0.89	0.08	1.34	0.35	6.23	3
Cu	8.65	63.97	12.98	95.96	60.55	447.79	30
Hg	<0.01	0.03	<0.015	0.05	<0.07	0.21	1
Li	<0.25	0.76	<0.375	1.14	<1.75	5.32	25
Mo	2.11	9.93	3.17	14.90	14.77	69.51	10
Ni	0.08	4.69	0.12	7.04	0.56	32.83	5
Pb	<0.05	0.24	<0.075	0.36	<0.35	1.68	5
Sb	<0.20	<0.20	<0.3	<0.3	<1.4	<1.4	20
Se	<1.30	<1.30	<1.95	<1.95	<9.1	<9.1	130
Sn	<0.60	<0.60	<0.9	<0.9	<4.2	<4.2	60
Tl	<0.08	<0.08	<0.12	<0.12	<0.56	<0.56	8
V	<0.01	0.36	<0.015	0.54	<0.07	2.52	1

^a^ Results reported in Health Canada (2025).

^b^ 50th percentile reported daily/almost daily use for medical purposes (Government of Canada, 2023; Government of Canada, 2024).

^c^ 95th percentile reported daily/almost daily use for medical purposes (Government of Canada, 2023; Government of Canada, 2024).

^d^ European Pharmacopoeia 5.20 permitted daily exposures.

For cannabis vaping products, consumption was reported as number of puffs in the 2023 and 2024 CCS. To our knowledge, there are no comprehensive published studies that definitively provide ranges of vaping liquid consumed per puff, so the estimate of 5 mg/puff was used, based on online sources that provide expected ranges of how many puffs a vaping cartridge may yield (HelloMD, 2018; Vaping360, 2025). This resulted in a range of approximately 3–6 mg/puff. From this, the highest typical amounts for daily or almost daily vape consumers were among those who consumed cannabis for non-medical purposes, at 0.05 g/day (based on a median of 10 puffs/day) and this value was used for risk assessment of vaping liquids (Table 2); medical consumers consumed a median of 7 puffs/day. The 5 mg/puff estimate and 0.05 g/day assumption align with a study by Reed et al. (2024), where the estimated average THC delivered in a vaping session is about 40 mg (0.05 g of an 80% THC vape would be 40 mg THC). Heavy use was identified as those who consumed 0.3 g/d based on the 95th percentile (60 puffs); this value was the same among daily/almost daily consumers for both medical and non-medical purposes.

**TABLE 2:** Estimated daily exposure to metals from typical or heavy daily/almost daily cannabis vaping product use. Shaded cells indicate where a European Pharmacopoeia permitted daily exposure or derived daily exposure limit would be exceeded.

Metals	Reported metal concentrations^a^ (µg/g)		Estimated daily exposure (µg/d)				Daily exposure limits^d^ (µg/d)
	Median	Maximum	Typical^b^ daily use (0.05 g) with median metal concentration	Typical^b^ daily use (0.05 g) with maximum metal concentration	Heavy^c^ use (0.3 g) with median metal concentration	Heavy^c^ use (0.3 g) with maximum metal concentration	
As	0.0062	0.1000	0.0003	0.0050	0.0019	0.0300	2
Cd	<0.0054*	0.0077	<0.0003	0.0004	<0.0016	0.0023	3
Co	0.1900	0.9600	0.0095	0.0480	0.0570	0.2880	3
Cr	0.2940	21.7000	0.0147	1.0850	0.0882	6.5100	3
Cu	0.5690	485.0000	0.0285	24.2500	0.1707	145.5000	30
Fe	1.6940	84.8300	0.0847	4.2415	0.5082	25.4490	41,500
Hg	<0.015*	0.0278	<0.0008	0.0014	<0.0045	0.0083	1
Mn	0.0400	1.8800	0.0020	0.0940	0.0120	0.5640	6
Ni	0.5210	54.7000	0.0261	2.7350	0.1563	16.4100	5
Pb	0.0281	0.6280	0.0014	0.0314	0.0084	0.1884	5
V	0.0050	0.5400	0.0003	0.0270	0.0015	0.1620	1
Zn	1.5320	310.0000	0.0766	15.5000	0.4596	93.0000	41,500

^a^ Results reported in Gajdosechova et al. (2022).

^b^ European Pharmacopoeia 5.20 permitted daily exposures, except Fe, Mn and Zn, which were derived from minimum risk levels (Mn) or recommended exposure limits (Fe and Zn).

^c^ 50^th^ percentile reported daily/almost daily use for non-medical purposes (Government of Canada, 2023; Government of Canada, 2024).

^d^ 95^th^ percentile reported daily/almost daily use for medical and non-medical purposes (Government of Canada, 2023; Government of Canada, 2024).

Unfortunately it is not possible to tie the consumption amounts taken from the surveys to specific THC doses with a great amount of certainty, due to some of the inherent limitations of the data from the CCS, however some assumptions can be made regarding the associated THC doses. Most dried cannabis products sold in Canada are above 200 mg/g total THC, with many at or approaching 300 mg/g. However, it has been noted that THC labels overestimate what any particular package of dried cannabis may contain (e.g., Brown, 2023; Health Canada, 2025). If we assume 250 mg/g of total THC as a typical dried cannabis product in Canada, then typical daily consumers would be presumed to be exposed to 375 mg of THC and 1750 mg for heavy use. However, most of this THC is expected to be lost to pyrolysis and side stream smoke (Gieringer et al., 2004; Elzinga et al., 2014; Sheehan et al., 2019; Cabecinha et al., 2025). Vaping products sold in Canada are typically 700–900 mg/g total THC, and if we assume 800 mg/g, total THC exposure in daily cannabis vapers would be 40 mg and 240 mg THC for typical and heavy use. Having defined THC doses can help in risk assessment, as the specific amounts of total THC in a product can be used in exposure assessment for product-specific health risk assessment.

With the aforementioned consumption amounts, median or maximum concentrations (µg/g) for the measured metals were used to estimate typical and a worst-case scenario daily metal exposure (µg/d). These were compared to Ph Eur PDEs or to derived daily exposure limits adapted from other inhaled metal exposure limits when PDEs were not established. The rationale for choosing the maximum levels measured was to support any actions necessary to mitigate risk to consumers exposed to those particular lots of cannabis product.

## 3 Challenges in risk assessment

### 3.1 Pharmacopoeial standards are applied in the Canadian regulatory requirements for cannabis

The *Cannabis Regulations* do not list specific metals with specific limits, but reference standards found in pharmacopoeias, an approach consistent with that used for drugs. Unlike drugs, however, in Canada there is no pre-market review or approval to verify or validate how producers conduct their testing on cannabis products. Our current understanding of industry practices is that testing is often limited to the Class 1 metals As, Cd, Hg and Pb while the elemental impurity standard in the pharmacopoeias list 24 heavy metals. The standard provides guidance on how a manufacturer should conduct a risk assessment in order to determine when each metal should be tested for and to what specifications, in line with expectations or common practices of developing robust contaminant preventive control plans (PCPs). As such, compliant GPPs and PCPs would be expected to consider more than simply the Class 1 metals, and test for and limit other metal impurities to reasonably achievable levels when there is the possibility they could contaminate a product, and not simply to the highest concentration that would avoid crossing a PDE threshold. The challenge for producers is to correctly understand and apply the pharmacopoeial standard and its nuances in order to clearly demonstrate compliant practices.

### 3.2 Applying permitted daily exposures

PDEs are health-based exposure limits that can be compared with the observed or predicted levels of impurity exposure (Ball and Beierschmitt, 2020). The PDE represents a substance-specific dose that is unlikely to cause an adverse effect if an individual is exposed at or below this dose every day for a lifetime, with different PDEs established for the different routes of administration (International Council for Harmonisation, 2022). The PDE is generally derived from the longest duration animal study, represents general systemic toxicity and uses several modifying factors for human extrapolation. It is considered to be protective of health for all patient populations. One key limitation of using the PDE for cannabis products is that inhalational PDEs were developed for unheated products (e.g., that use metered dose inhalers), without additional considerations for smoked or vaped products. There may be differences in exposure and/or toxicity due to heating, resulting in preferential partitioning of the metal impurities in smoke or vapour streams. Moreover, nano-sized metal particles have been reported in cannabis vaping product liquids (Gajdosechova et al., 2025; Gajdosechova and Marleau-Gillette, 2024). Applicability of PDEs to metal nanoparticles needs further investigation due to the reactivity and increased deposition and penetrative capacity of nano-sized particles in the lung.

Since PDEs represent safe long-term exposure, they can be considered protective against acute or short-term effects. This is because in general, the dose required to elicit acute or sub-chronic effects is several-fold above the chronic dose. However, PDEs do not represent the toxicology endpoint of irritation or sensitization.

For inhaled cannabis products, the effects of THC on the consumer will self-limit product exposure to levels where typical metal impurity concentrations are not anticipated to elicit acute effects. For example, unlike e-cigarettes, whose consumption can be as high as 1–10 mL/d (Fowles et al., 2020), cannabis vaping liquids are usually consumed in relatively small quantities (Government of Canada, 2023; Government of Canada, 2024) due to the doses of THC involved and consequent psychoactive or other noxious effects that self-limit consumption. Thus, acute metal toxicity is unlikely and risk assessment should consider long-term exposure, where PDE limits are deemed more appropriate health risk benchmarks.

Besides the risk-based approach for limiting absolute daily exposure (µg/d), there is also a quality-based concentration limit approach. The latter is widely used by the industry and cannabis regulatory bodies in the U.S., where the concentration limit used corresponds to products where no more than 10 g would be consumed per day. Limiting metal impurities to these concentration limits provides a safety margin for dried cannabis and cannabis vapes since it is unlikely or rare that more than 10 g would be consumed on a daily basis (Government of Canada, 2023; Government of Canada, 2024).

#### 3.2.1 What if there is no PDE value?

PDE limits have not been established in the International Council for Harmonization guideline for iron (Fe), manganese (Mn) and zinc (Zn). These have low inherent toxicity and were observed in the study data. In such cases, derived daily exposure limits from other regulatory agencies can be applied. However, expert judgement is required when extrapolating values in this way, especially when risk assessors must use exposure limits that have a completely different exposure paradigm in occupational or environmental settings. Such concentration limits should be converted to absolute exposures and not used directly. Previous studies have detailed this approach (Farsalinos et al., 2015; Farsalinos and Rodu, 2018; McDaniel et al., 2021; Soulet and Sussman, 2022; Vreeke et al., 2022). For example, the minimal risk level (MRL) for Mn (0.3 μg/m^3^) or recommended exposure limits (RELs) for Fe and Zn (5000 μg/m^3^) can be converted into total daily exposures using an inhalation volume of 20 m^3^ (for 24 h) and 8.3 m^3^ (for 10 h), respectively (derived values are listed in Table 2). The MRL to be applied is defined by the Agency for Toxic Substances and Disease Registry (2025), while the REL is defined by the National Institute of Occupational Safety and Health (Centers for Disease Control and Prevention, 2019a; Centers for Disease Control and Prevention, 2019b) (see calculations in the Supplementary Materials S2).

## 4 Exposure assessment

Exposure assessment is the process that involves producing a qualitative and/or quantitative estimate of the magnitude, frequency, duration, route and extent of human exposure to an agent (Health Canada, 2000). It also specifies the target population and sensitive sub-population. In our case study on metals, the target population used is consumers who use cannabis on a daily or almost daily basis and the sensitive sub-population of highest concern is people with medical impairments, such as metabolic disorders or compromised lung function, that increase vulnerability to the toxic effects of inhaled metals, for example Cu toxicity for those with Wilson’s disease (Roberts and Schilsky, 2008).

The biggest challenge in cannabis risk assessment is estimating the systemic exposure dose. In this case, we derived daily exposure estimates for those who may be using the product on a daily basis, as those consumers would be the population for which a PDE would be most pertinent to compare against. According to the 2023 and 2024 cycles of the CCS, 7.9% of Canadians 16 and older reported daily or almost daily use. Of these, 6.0% reported using for non-medical purposes and 3.2% for medical purposes, with some overlap of respondents saying they used for both. For the purposes of this assessment, only 2023 and 2024 pooled data were used due to methodological changes to survey exposure questions from 2022 and earlier. Medians were used for typical use, since some responses (e.g., 1,000 puffs per day for vapes) skewed mean values.

The median amount consumed on a typical use day for daily or almost daily cannabis consumers was 1.5 g for dried cannabis and 0.05 g (10 puffs) from a vape pen/cartridge, while for heavy use it was 7.0 g and 0.3 g (60 puffs) for dried cannabis and a vape pen/cartridge, respectively. The heavy use amount compounds with the maximum measured metal values and 100% transfer efficiency assumption, representing an extreme exposure scenario, from which it would not be appropriate to draw risk assessment conclusions with certainty. Since well-defined transfer rates of metals to cannabis product emissions do not yet exist, using measured values from dried cannabis or vape liquids will overestimate exposure.

Even with daily use amounts defined for particular product types, uncertainty regarding the systemic exposure dose is introduced by different consumer behaviours. Puff volume, puff duration, breath-hold duration and heating method, among other variables, can vary significantly between users (Azorlosa et al., 1995; Bidwell et al., 2018; Government of Canada, 2024), introducing additional uncertainty in risk assessment.

## 5 Risk characterization

Only a small proportion of Canadians consume dried cannabis or cannabis vaping liquids daily or almost daily, thus the observed metal levels in this case study do not present an elevated risk to the majority of cannabis consumers. However, there may be elevated risk to individuals more susceptible to inhaled metal toxicity.

As seen in Tables 1, 2, for daily consumers, risk is characterized as low when the estimated daily exposure of the metal impurity is below its PDE (or derived limit when a PDE was not present). Assuming 100% metal transfer to emissions and absorption, none of the Class 1 metals exceeded the PDEs in the case study for either dried cannabis or vaping liquids. Furthermore, at the median study values, typical daily use did not exceed the PDEs or derived limits for any metals for either vaping liquids or dried cannabis. However, for dried cannabis, typical daily use at the maximum observed metal levels exceeded the PDE values for Cu, Mo and Ni. Furthermore, daily heavy use of dried cannabis at the median metal levels exceeded PDEs for Cu and Mo and daily heavy use at the maximum observed metal levels exceeded the PDEs for Cr, Cu and Ni in vape liquids and for Cr, Cu, Mo, Ni and V in dried cannabis. In the case of heavy use of dried cannabis at the median metal level, we cannot attribute a high level of concern for Mo and Cu without more certainty around the overestimated exposure from 100% transfer to emissions, inhalation and absorption and even then, we must recognize that these are both essential human trace elements readily dealt with by the body.

Do the metals exceeding PDEs raise a health risk concern warranting additional regulatory action? Not for most Canadians, since they are not daily cannabis consumers. However, a comprehensive toxicology review, including comparisons with regulatory limits from other regulatory bodies, is required as toxicity has not yet been characterized specifically for the context of cannabis use. More emissions data are also required to facilitate exposure assessments that may better reflect real-life exposure with more certainty around the current assumptions (such as 100% metal transfer to emissions and absorption) that overestimate exposure.

For a general understanding of the risk profile that dried cannabis and cannabis vaping products pose, typical daily use at the median metal level ideally represents the appropriate cannabis-user profile for risk assessment, while heavy use with median metal levels is considered as a worst-case scenario. Using maximum metal levels with heavy use further exaggerates the worst-case scenario and does not translate to realistic cannabis product risk profiles. While this risk assessment considered the highest measured levels of metals in order to inform regulatory actions to mitigate risk to those consumers exposed to those specific products, for a more generalized understanding of risk that these types of products pose, it would be more appropriate to use the 90th or 95th percentile metal levels. When the 95th percentile metal levels were used, the only PDEs that were exceeded were for dried cannabis, where Cu exceeded the PDE for both typical and heavy daily use and Mo for heavy daily use only.

Furthermore, while the evidence-base on metal transfer, inhalation and absorption is still limited, and conclusions drawn from these data would still have a high degree of uncertainty, if other exposure assumptions are made, the level of risk concern may be reduced accordingly. For example, for dried cannabis, a 50% metal transfer to emissions and inhalation of 50% of the emissions (due to loss in side-stream smoke) would result in PDE limits being exceeded only for Cu, Mo and Ni (and not Cr or V) for daily heavy use at the maximum measured levels and only Cu and Mo at the 95th percentile measured levels. For vaping liquids, with the assumption of 25% transfer and inhalation of 100% of the emission (since there are no side-stream emissions when vaping) and daily heavy use at the maximum measured levels, only Cu (and not Cr or Ni) would exceed the PDE, and no metals would exceed the PDE using the 95th percentile measured levels. These assumptions can still be considered conservative in that they do not take into account what might be exhaled or ingested. The limited existing literature may support even lower levels of exposure, as it suggests rates of metal transfer to smoke and vape emissions are low (see Supplementary Materials S3 for an alternative exposure assessment based on these limited data).

## 6 Limitations and uncertainties

A number of uncertainties have been identified in the case study due to existing knowledge gaps.

Long-term daily use may increase health risks from metal impurities because of cumulative uninterrupted exposure, which may result in metal accumulation in the body or impair homeostatic or detoxification mechanisms and result in adverse health outcomes. Unlike long-term effects, acute effects (short-term health risk) are generally easier to identify as the time between exposure and effect is short; also, adverse effects may be mitigated with cessation (Staal et al., 2022). Frequent and shorter between-exposure interval patterns may also diminish the body’s ability to recover (Bos et al., 2021). Furthermore, inhaling high levels of cannabis emissions may compromise normal lung function over time, potentially altering an individual’s susceptibility to the risks of inhaled metals. More research is necessary to understand how each of these factors can impact the risk of inhaled metals from daily or almost daily cannabis inhalation.

Using the metal levels in the product as an internal as an internal dose overestimates exposure since levels were not measured in emissions. There are insufficient data to determine the transfer efficiency with certainty, and this may depend on the volatile nature of the metal, aerodynamic properties of the emissions (particle size), whether the metals are in particle or ionic form, speciation, device temperature and puff topography, among other factors. Additionally, for dried cannabis, exposure would be expected to be lower after deducting metals in side stream emissions, that are exhaled or that remain in the left-over joint, ash or filter.

Furthermore, metal impurity levels in cannabis products can vary from batch to batch and if a specification is not exceeded in every batch, then the PDE would only be exceeded for a short period of time (i.e., when the product being consumed is from a specific batch with higher levels of contamination) in daily consumers only. In reality, consumers are not exposed to a specific batch of product for many years or a lifetime.

While 100% lung deposition and absorption are also assumed, actual lung deposition is likely lower. It is understood that exhaled emissions may carry significant amounts of small particulate matter, which could include metals, and ingestion is also likely as particulates deposited in the mouth or throat may be swallowed.

The risk analysis did not include use of dried cannabis in vapourizers, simultaneous exposure to several metals, metal presence as nanoparticles or examine health-impaired individuals.

Although animal toxicity studies are well-defined based on exposure periods, human cannabis exposure periods are not well-defined. Typically, chronic exposure is often referred to in cannabis literature as weekly or more frequent use over months or years, while for chemical risk assessment it is continuous exposure over a long period of time (typically years, or even lifetime exposure in the case of PDEs).

## 7 Future directions and comments

Many unknowns impact the ability to perform accurate risk assessment of metals in smoked and vaped cannabis products. Standardized and well-understood vaping and smoking topographies for cannabis are much needed to characterize exposure. Studies measuring metals in aerosols or main-stream smoke emissions are required to establish their transfer efficiency as well as studies measuring their deposition when inhaled. Presence in the form of nanoparticles is another area of concern where more relevant risk metrics and standards need to be explored.

While it is unrealistic to expect absence of metal impurities in inhalable cannabis products due to unavoidable presence in the plant or leaching/contamination from vaping devices and product manufacturing, GPPs can limit their presence, in particular for Class 1 metals where the ability to reach very low concentrations is well-established. For Class 2 and 3 metals, additional data are still required to determine what quality management systems, such as compliant GPPs, are reasonably expected to achieve.

Due to the diversity of cannabis products, user profiles and use scenarios, risk analysis is more complicated than with therapeutic drug products, where there is well-defined dosing and where risk/benefit analyses can clearly establish concentration limits during pre-market review. Risk assessment is a dynamic process, and it will evolve as more data and better methods become available in characterizing risk to a greater certainty. Until the identified uncertainties in the risk assessment of metal impurities based on PDEs in smoked and vaped cannabis products are addressed, a quality-based approach of limiting metal levels to concentration limits that compliant GPPs are reasonably expected to achieve can mitigate risk to consumers. This risk analysis supports that the goal of the *Cannabis Act* to supply of adult-only access to a quality-controlled supply of cannabis is being achieved, as the typically seen metal concentrations are below levels that may present health risks to the vast majority of cannabis consumers.

## Data Availability

The data analyzed in this study is subject to the following licenses/restrictions: Metals results for cannabis vaping products are available in the supplementary information here: https://pubs.acs.org/doi/10.1021/acsomega.2c03797. Results for dried cannabis were taken from data tables generated by Health Canada’s Cannabis Data Gathering Program project. While the tables are not publicly available, the project details can be found here: https://www.canada.ca/en/health-canada/services/publications/healthy-living/data-gathering-program-comparison-legal-illegal-dried-cannabis-products.html. Canadian Cannabis Survey datasets used to characterize exposure are publicly available here: 2023: https://open.canada.ca/data/en/dataset/6c240c79-c857-4fd4-bbd6-b1fd7f04fdbc 2024: https://open.canada.ca/data/en/dataset/2abe0796-c3e6-443b-a5c5-74e770e9fbe0. Requests to access these datasets should be directed to AW, andrew.waye@hc-sc.gc.ca.
