# Supplementary material for: Risk assessment of metals measured in regulated Canadian dried cannabis and cannabis vaping products: case study and perspectives
Source: Front Toxicol. 2026 Jan 15;7:1755875. doi: 10.3389/ftox.2025.1755875 (PMC12851951; doi:10.3389/ftox.2025.1755875)
Supplement: Supplementary file 1 [file DataSheet1.pdf]

Supplementary Material

S1. Derivation of consumption amounts

Table S1. The 2023 and 2024 Canadian Cannabis Survey results, used to define typical (median) and heavy (95th percentile) daily/almost daily use amounts for exposure assessment. Total sample size: n=23,356 (2023: n=11,690; 2024: n=11,666).

	**Daily/almost daily use**
**Population prevalence**	**Proportion (n)**
Non-medical purposes (or both)	6.0% (1,466)
Medical purposes (or both)	3.2% (766)
Overall	7.9% (1932)
	**Daily/almost daily use (g)**
**Dried flower consumed on typical usage day (g)**	**50th, 95th percentile**
Non-medical purposes (or both)	1, 5
Medical purposes (or both)	1.5, 7
	**Daily/almost daily use**
**Vape pen/cartridge consumed on typical usage day (puffs)**	**50th, 95th percentile (puffs)**
Non-medical purposes (or both)	10, 60
Medical purposes (or both)	7, 60

S2. Calculation of exposure from MRL and REL

Adapted from Farsalinos and Rodu (2018).

For MRLs, the air concentration was used to calculate the 24-h daily exposure, based on the formula:

MRL 24-h exposure (µg) = MRL (µg/m^3) × ventilation volume in 24-h (m^3)

For RELs, a (10-h) daily exposure was assumed. Thus, the air concentration defined by RELs was used to calculate a 10-h daily exposure, based on the formula:

REL 10-h exposure (µg) = REL (µg/m^3) × (ventilation volume in 10-h)

The European Medicines Agency uses an average daily inhaled air volume of 20 m^3/day (European Medicines Agency, 2008).We used this, which is more conservative; thus, the 24-h inhalation

volume was set at 20 m^3 (for MRLs) and the 10-h inhalation volume was calculated at 8.33 m^3 (for RELs).

S3. Alternative exposure assessment based on existing limited data

S3.1 For dried cannabis: Cu, Cr, Ni, Mo and V

In a study characterizing cannabis smoke (Moir et al., 2008), metals like Cd, Pb, Cr and Ni, were present in both mainstream and side-stream cannabis smoke, with higher levels in side-stream smoke (several fold). While smoking, the consumer is directly exposed to mainstream smoke only (smoke inhaled into mouth); consequently, a low inhalation dose can be conservatively assumed to be 50%.

Besides mainstream smoke, the transfer efficiency of metals from dried cannabis to smoke is considered. Transfer rate varies greatly depending on the metal and its volatility, and available information from tobacco smoke when extrapolated to cannabis indicates low transfer efficiency. The metal transfer efficiencies reported for tobacco cigarettes are as low as Cr 0.7-8.3%, Ni 3.6-15.5% (Pinto et al., 2017) and Cu 1.7% (Cogbill and Hobbs, 1957). Transfer efficiency of Mo and V is not available but is likely to be similarly low. The physical characteristics of aerosol particles produced by smoking tobacco cigarettes or cannabis joints have been reported to be qualitatively similar with quantitative differences in size, mass and chemical composition (Graves et al., 2020). A 50% transfer efficiency can be conservatively assumed for the metals that exceeded the PDE in smoke.

By refining exposure assessment with 50% inhalable dose and a transfer efficiency of 50%, PDEs were exceeded only for Cu, Mo and Ni but only under heavy use conditions and using the maximum level metal values.

S3.2 For vape liquid: Cr, Cu and Ni

The metal transport efficiencies from electronic nicotine delivery system liquid to aerosol were, in general, quite low, averaging less than 1% to 4.7% (Halstead et al., 2020). Since the limited evidence suggests much lower transfer efficiencies than above for smoked cigarettes, a 25% transfer efficiency

can be used and considered conservative, with no side stream loss. In this case, PDE would only be exceeded for Cu under heavy use at the maximum level metal value for Cu.

S4. References for Supplementary Material document

Cogbill, E. C., and Hobbs, M. E. (1957). Transfer of metallic constituents of cigarettes to the mainstream smoke. Tob. Sci. 1 (15), 68–73.

European Medicines Agency. (2008). Guideline on the specification limits for residues of metal catalysts or metal reagents. guideline-specification-limits-residues-metal-catalysts-or-metal-reagents_en.pdf

Graves, B. M., Johnson, T. J., Nishida, R. T., Dias, R. P., Savareear, B., Harynuk, J. J., et al. (2020). Comprehensive characterization of mainstream marijuana and tobacco smoke. Sci. Rep. 10 (1), 7160. doi:10.1038/s41598-020-63120-6

Halstead, M., Gray, N., Gonzalez-Jimenez, N., Fresquez, M., Valentin-Blasini, L., Watson, C., et al. (2020). Analysis of toxic metals in electronic cigarette aerosols using a novel trap design. J. Anal. Toxicol. 44 (2), 149–155. doi:10.1093/jat/bkz078

Moir, D., Rickert, W. S., Levasseur, G., Larose, Y., Maertens, R., White, P., et al. (2008). Comparison of mainstream and sidestream marijuana and tobacco cigarette smoke produced under two machine smoking conditions. Chem. Res. Toxicol. 21 (2), 494–502. doi:10.1021/tx700275p

Pinto, E., Cruz, M., Ramos, P., Santos, A., and Almeida, A. (2017). Metals transfer from tobacco to cigarette smoke: evidences in smokers' lung tissue. Environ. Pollut. 231, 110–118. doi:10.1016/j.jhazmat.2016.11.069
